# Case Report: Telitacicept in the treatment of cSLE-APS: novel therapeutic perspectives on autoimmune thrombotic diseases in children

**DOI:** 10.3389/fimmu.2025.1675554

**Published:** 2026-02-06

**Authors:** Jingyue Liu, Zhilang Cao, Yajun Wang, Jing Zheng

**Affiliations:** 1 Department of Pediatrics, The First People’s Hospital of Yunnan Province, Kunming, China; 2 The Affiliated Hospital of Kunming University of Science and Technology, Kunming, China

**Keywords:** antiphospholipid syndrome, B-cell, childhood-onset systemic lupus erythematosus, telitacicept, thrombotic event

## Abstract

Thrombotic antiphospholipid syndrome (APS) in Childhood-onset systemic lupus erythematosus (cSLE) remains a therapeutic frontier. Here, we report the first case of telitacicept (a TACI-Fc fusion protein)- induced remission in cSLE related antiphospholipid syndrome (cSLE-APS), where targeted dual BAFF(B cell-activating factor)/APRIL(a proliferation-inducing ligand) inhibition resolved life-threatening infarctions and achieved sustained disease control. During treatment, corticosteroids were successfully discontinued within six months, and a lupus low disease activity state (LLDAS) was maintained. Our findings indicate that telitacicept’s unique ability to regulate B-cell activity could target the dual pathology of cSLE-APS, offering a new therapeutic approach for this serious condition.

## Introduction

Childhood-onset systemic lupus erythematosus (cSLE) is a multisystem autoimmune disorder characterized by heterogeneous clinical manifestations and diverse autoantibody production. Compared to adult-onset SLE, cSLE demonstrates more aggressive disease features, including higher rates of severe renal, neurological, and hematological involvement, increased disease activity, greater cumulative organ damage, and elevated morbidity and mortality (1).

Antiphospholipid antibodies (aPL) are detected in 20%-40% of patients with SLE (2). Among these aPL-positive individuals, a substantial proportion (reported in up to 50-70% in some cohorts) may progress to overt antiphospholipid syndrome (APS) (3). In the general population, APS is a rare disease with an estimated incidence of approximately 2 cases per 100,000 person-years (4). The aPL profile includes lupus anticoagulant (LA), anticardiolipin antibodies (aCL), and anti-β2-glycoprotein I antibodies (aβ_2_GPI). APS, it is no longer defined by “recurrent thrombosis and obstetric morbidity” only-Other non-thrombotic manifestations have been included in the wide clinical spectrum in the 2023 ACR/EULAR classification criteria, such as aPL Nephropathy, thrombocytopenia, etc (5). APS is classified as primary or secondary—the latter being more prevalent and strongly associated with SLE (6). Systemic lupus erythematosus related antiphospholipid syndrome (SLE-APS) exhibits a particularly severe phenotype, characterized by heightened organ damage, morbidity, and mortality compared to primary APS (7). Current management of SLE-APS emphasizes anticoagulation as the cornerstone therapy, while refractory cases may require adjunctive glucocorticoids and immunosuppressants. Emerging evidence suggests potential roles for biologics such as rituximab (8, 9), though robust clinical data, especially for cSLE-APS remain limited.

We present a cSLE-APS case initially manifesting as autoimmune hemolytic anemia (AIHA), later complicated by myocardial and cerebral infarction. Following combination therapy with telitacicept(a novel recombinant transmembrane activator and calcium modulator and cyclophilin ligand interactor (TACI)-Fc fusion protein targeting BAFF and APRIL) (10), glucocorticoids, and immunosuppressants, the patient achieved lupus low disease activity state (LLDAS), sustained remission, and successful glucocorticoid withdrawal within six months, without recurrent thrombosis. This case highlights the potential of targeted biologic therapy for rapid disease control and durable remission in cSLE-APS.

## Case description

An 8-year-old female patient presented to our hospital in August 2023 with a 2-year and 9-month history of recurrent jaundice and fatigue. Initial evaluation at a local hospital revealed anemia and a positive direct Coombs test, leading to a diagnosis of AIHA. Treatment with oral prednisone (40 mg daily) was initiated, and it was subsequently tapered to 5 mg daily. During the steroid tapering, the patient developed dizziness, gait instability, and slurred speech, prompting re-evaluation. Further laboratory investigations revealed a positive antinuclear antibody (ANA) titer (1:320), low levels of complement 3(C3), and positivity for aCL and LA. Cardiac imaging showed segmental hypokinesis of the left ventricular wall, an echo-dense mass in the left atrial appendage (suggestive of thrombus), pulmonary artery dilation, and possible myocardial infarction in the inferior and posterior walls of the left ventricle. Cardiac magnetic resonance imaging(MRI) confirmed the left atrial appendage thrombus and suspected myocardial infarction.

Brain MRI imaging detected signal abnormalities in the right frontal lobe and bilateral parietal lobes, consistent with cerebral infarcts. The patient was diagnosed with autoimmune disorders, including suspected APS and SLE. Treatment included rituximab (375 mg/m² weekly for 4 doses), prednisone, hydroxychloroquine, clopidogrel bisulfate, and warfarin anticoagulation. After treatment, the child continues to show significant symptoms of heart failure, classified as New York Heart Association (NYHA) functional class III.

**Figure 1:**
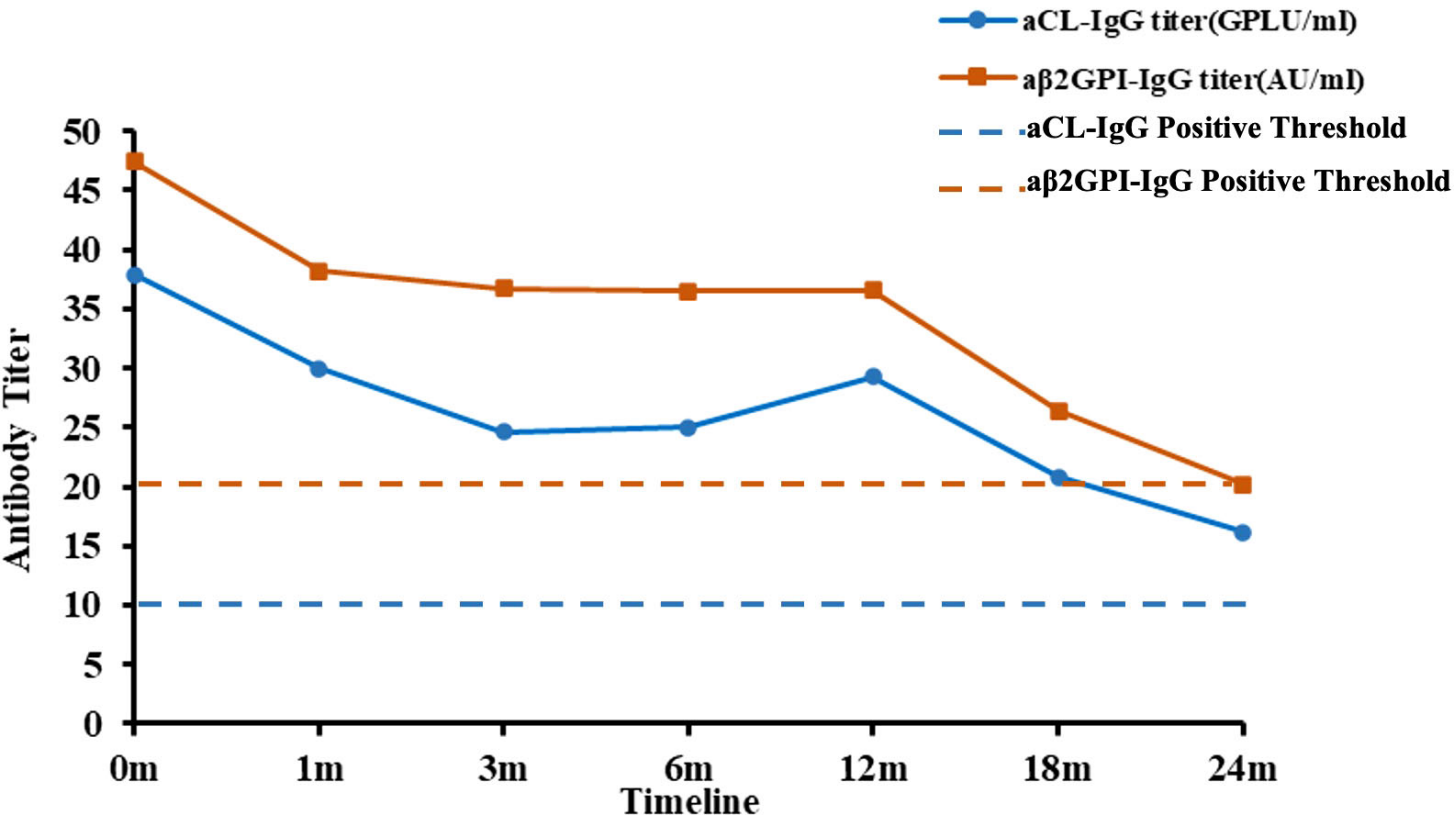
Trends in aCL-IgG and aβ2GPI-IgG titer. m, month; aCL-IgG: Anticardiolipin antibody of IgG; aβ2GPI-IgG: Anti-β2-glycoprotein-I antibody. Laboratory reference ranges: aCL-IgG titer: 0–10 GPLU/ml; aβ2GPI-IgG titer: 0–20 AU/ml.

**Figure 2:**
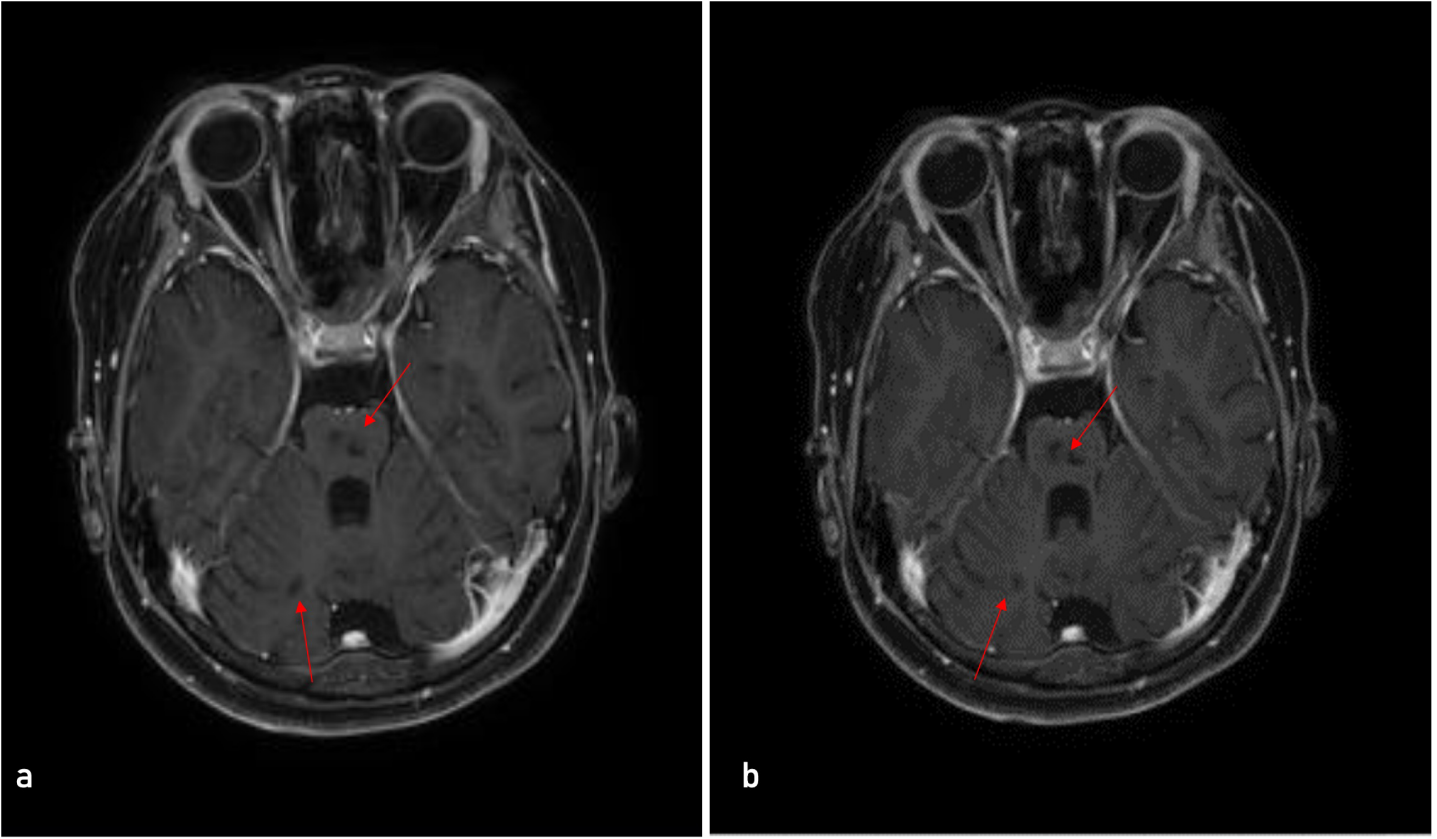
Serial axial T2-weighted images showing multiple chronic ischemic lesions with encephalomalacia. **(a)** Image at one level demonstrates the lesions in the bilateral cerebellar hemispheres and the pons (the area marked by the red arrow); **(b)** An adjacent image at another level confirms the presence and extent of the aforementioned lesions (the area marked by the red arrow).

Our hospital laboratory and imaging findings: complete blood count(CBC) revealed leukocytosis (13.49×10^9/L) with neutrophils 8.36×10^9/L, lymphocytes 4.04×10^9/L, hemoglobin 154 g/L, and platelets 325×10^9/L. C-reactive protein was <0.5 mg/L, and erythrocyte sedimentation rate was 2 mm/h. Lymphocyte subset analysis showed a CD4/CD8 ratio of 0.76 and CD19+ B cells at 0.2%. Autoantibody testing was negative for antinuclear antibodies (ANA), anti-Smith antibodies, double-stranded DNA (dsDNA) antibodies, and antineutrophil cytoplasmic antibodies (ANCA). However, initial and follow-up testing confirmed persistent positivity for LA and the “IgG isotypes” of both anticardiolipin aCL and aβ_2_GPI antibodies. The detectable IgG aPL titers at baseline and their subsequent decline over 24 months of treatment are shown in **Figure 1**. Complement protein levels were slightly reduced: C3 0.87 g/L (reference range: 0.9–1.8 g/L) and C4 0.17 g/L (0.1–0.4 g/L). Direct Coombs test was positive. Cardiac biomarkers demonstrated elevated B-type natriuretic peptide (BNP) at 479.4 pg/mL (normal: 0–96.16 pg/mL), while creatine kinase-MB (CK-MB), myoglobin, and troponin levels were within normal limits. Coagulation studies revealed prolonged activated partial thromboplastin time (APTT: 50.3 s; normal range: 28–43.5s) and prothrombin time (PT: 32.5 s; normal range: 11–15 s). Urinalysis revealed no abnormalities. The echocardiography findings indicate mild to moderate left ventricular enlargement with hypokinesis primarily affecting the posterior wall. There is evidence of both systolic and diastolic dysfunction in the left ventricle, with the ejection fraction (EF) measured at 49%. Mild regurgitation was observed in the aortic, mitral, and tricuspid valves, alongside reduced flow velocities across all cardiac valves. A small pericardial effusion was present. NYHA functional class III. The brain MRI revealed multiple chronic ischemic lesions accompanied by partial encephalomalacia in both the bilateral cerebellar hemispheres and the pons (**Figure 2**). These findings are consistent with remote infarcts, indicating prior ischemic events in these regions.

**Figure 3:**
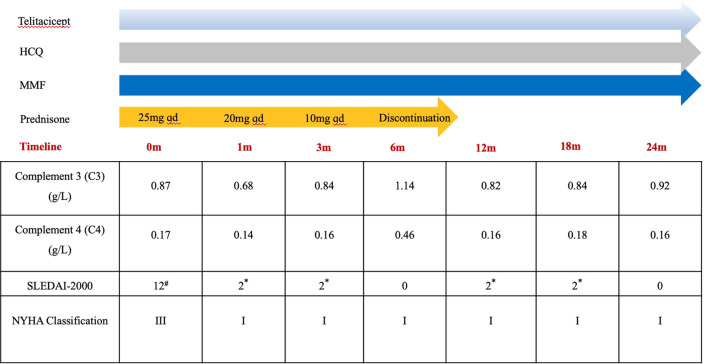
Treatment timeline and key laboratory parameter changes. 12^#^(encephalopathy, pericarditishy and hypocomplementemia). 2* (hypocomplementemia) m, month; HCQ, Hydroxychloroquine; MMF, Mycophenolate Mofetil; NYHA, New York Heart Association; SLEDAI-2000, Systemic Lupus Erythematosus Disease Activity Index. Laboratory reference ranges: C3:0.9-1.8g/L; C4:0.1-0.4 g/L.

Based on the 2019 European League Against Rheumatism/American College of Rheumatology(EULAR/ACR) classification criteria for SLE (11), the patient scored 16 points for SLE diagnosis and was definitively diagnosed with systemic lupus erythematosus and secondary antiphospholipid syndrome. The Systemic Lupus Erythematosus Disease Activity Index 2000 (SLEDAI-2000) was 12 points, indicating moderate activity. Given the involvement of vital organs in this pediatric patient, the treatment regimen was modified as follows: Telitacicept was added to modulate immune responses(80 mg weekly), combined with Mycophenolate Mofetil for enhanced immunosuppression, while Hydroxychloroquine was continued as maintenance therapy. The dose of Prednisone was adjusted to 25mg/day(1mg/kg/day) to control inflammatory activity, and Digoxin along with Spironolactone were administered to improve cardiac function (**Figure 3**).

The patient was followed for a total of 24 months, from August 2023 to August 2025. Disease activity was serially assessed using the validated SLEDAI-2000 (12). Key clinical and laboratory assessments were performed at baseline, 1, 3, 6, 12,18 and 24 months after initiating telitacicept therapy. As shown in **Figure 3**, telitacicept treatment led to a rapid and significant improvement in disease activity, with the SLEDAI-2000 score decreasing from 12 at baseline to 0 after 24 months of therapy. Throughout follow-up, prednisone was tapered off and discontinued within six months after telitacicept treatment. The patient maintained a LLDAS, showed decreasing antiphospholipid antibody titers (**Figure 1**), had normalized cardiac function, and experienced no new thromboembolic events. The late gadolinium enhancement (LGE) cardiac MRI and coronary angiography were not performed during the acute management. Throughout the telitacicept treatment and follow-up period, the patient was rigorously monitored for potential adverse events. Safety assessments were performed at each clinical visit, which coincided with the efficacy evaluations. The monitoring protocol included:(1)Clinical evaluation: Thorough physical examinations with particular attention to signs of infection (e.g, fever, cough), injection-site reactions (e.g., erythema, swelling, pain), and any new or worsening symptoms;(2) Laboratory investigations: Serial measurements of CBC, comprehensive metabolic panel (including liver and renal function tests), and immunoglobulin (IgG, IgA, IgM) levels;(3) Infection surveillance: Patients and their guardians were counseled to report any signs of infection immediately. Any episode of infection was documented in detail, including its type, severity, and management. No adverse events attributable to telitacicept, such as infections, cytopenias, or infusion-site reactions, were observed throughout the 24-month follow-up period.

## Discussion

The management of cSLE-APS presents a formidable clinical challenge, distinct from its adult counterpart. While the prevalence of APS in the pediatric SLE population is considerably lower—estimated at approximately 4% (13) compared to about 36.2% in adults (3)—its clinical impact is often more severe. cSLE-APS is characterized by a higher frequency of atypical and severe thrombotic manifestations and multisystem involvement, including a heightened risk of recurrent thrombotic events, particularly affecting the central nervous system (14). A large multicenter cohort study of 1,519 cSLE patients underscored that the coexistence of APS is a critical determinant of cumulative organ damage, with neuropsychiatric involvement being a hallmark and leading cause of morbidity (13). The cardiac presentation of this child—myocardial infarction in a young individual without traditional risk factors—strongly suggests APS-related microvascular pathology as a cause of myocardial infarction with non-obstructive coronary arteries(MINOCA) (15). Although LGE sequencing and coronary angiography were not performed, the combination of multi-system thrombosis, persistent high-titer aPL, and segmental wall motion abnormalities aligns with APS-mediated microvascular thrombosis and endothelial dysfunction. This highlights the need to consider MINOCA in cSLE-APS patients with cardiac symptoms. Future management of similar cases may benefit from advanced imaging, including LGE-MRI, to better characterize myocardial injury.

Furthermore, the therapeutic landscape for cSLE-APS is notably constrained. The lack of prospective randomized controlled trials specific to the pediatric population forces clinicians to extrapolate from adult guidelines, which presents significant therapeutic challenges (16). The management of cSLE-APS involves considering both SLE and APS at the same time. The cornerstone therapy of long-term anticoagulation, while essential, does not target the underlying autoimmune driver of the disease—the aberrant B-cell activation and pathogenic autoantibody production. This significant unmet medical need underscores the imperative for targeted biologic therapies that can effectively modulate the core immune dysfunction in cSLE-APS.

The initial serological profile of our patient—ANA positivity without anti-dsDNA—also raises the possibility of “systemic APS” a clinical entity where patients exhibit systemic inflammation and positive aPL but may not meet full SLE criteria. Recent cohort data (e.g., from the APS ACTION registry) indicate that only about 10% of such patients progress to classified SLE. This distinction is therapeutically relevant, as management of systemic APS” may center more on thromboprophylaxis, whereas clear SLE-APS necessitates combined immunosuppression (17). In our case, the subsequent fulfillment of SLE classification criteria and high disease activity (SLEDAI-2K=12) justified the use of a regimen targeting both inflammatory and thrombotic pathways.

The pathogenesis of APS involves CD20-negative B-cell subsets that drive pathogenic autoantibody production through aberrant differentiation (18).B-cell dysregulation—particularly in generation, proliferation, and plasma cell differentiation—directly influences aPL titers and disease severity in SLE-APS (19). B-cell activating factor (BAFF) and a proliferation-inducing ligand (APRIL) are pivotal regulators of B lymphocyte homeostasis: BAFF mediates the differentiation and maturation of immature B cells, while APRIL sustains the survival and function of long-lived plasma cells (LLPCs) (20). Elevated BAFF levels are observed in both primary APS and SLE-APS (21), supporting therapeutic targeting of these pathways.

Belimumab, the first biologic agent approved by the Food and Drug Administration(FDA) for the treatment of active SLE, exerts its therapeutic effects by blocking the binding of soluble BAFF to B-cell surface receptors, thereby inducing apoptosis of autoreactive B cells and reducing serum autoantibody levels. It has been reported that after treatment with belimumab for SLE-APS/primary APS, the titer of aPL antibodies decreased (22, 23). Recent work by Yannick et al. further demonstrates that in APS patients, phospholipid-reactive B cells—derived from polyreactive naïve B cells—escape peripheral tolerance and differentiate into LLPCs under interferon and APRIL-mediated signaling. These LLPCs continuously secrete aPL for an extended period in the bone marrow. This mechanistic insight underscores the rationale for dual BAFF/APRIL inhibition. Telitacicept, a novel fusion protein targeting both pathways, extends beyond belimumab’s scope by directly suppressing plasma cell-mediated antibody production (10). Although one adult SLE-APS study reported telitacicept’s efficacy in reducing aPL titers and thrombotic risk (24), its application in cSLE-APS remains unexplored.

The clinical and serological course of our patient provides tangible support for this mechanistic model. The dual inhibition of BAFF and APRIL by telitacicept likely underpins the profound B-cell and plasma cell modulation we observed. Specifically, the rapid and sustained reduction in aPL titers in our patient aligns with the suppression of APRIL-driven LLPCs, which are responsible for persistent autoantibody production. Concurrently, the achievement of an LLDAS can be attributed to the concomitant blockade of BAFF-mediated B-cell survival and maturation. Therefore, the favorable clinical and serological response observed in our case provides preliminary clinical support for the theoretical advantage of dual BAFF/APRIL inhibition in SLE-APS. It is important to note that BAFF inhibition alone (e.g., with belimumab) has also been reported to reduce aPL titers (22, 23). Our findings suggest that dual inhibition, by targeting a broader spectrum of B-cell dysregulation, represents a promising alternative therapeutic strategy worthy of further exploration in refractory cSLE-APS.

The safety profile of any biologic agent is a paramount consideration, especially in the pediatric population. When positioned against other biologics explored for SLE-APS, telitacicept presents a distinct risk-benefit profile. Rituximab, an anti-CD20 monoclonal antibody, leads to profound B-cell depletion and is associated with significant risks, including higher rates of infusion-related reactions, prolonged hypogammaglobulinemia, and an increased susceptibility to infections, which is a particular concern in patients already receiving immunosuppressants (25). Belimumab has a well-established and generally favorable safety profile in both adults and children with SLE. However, its most common concerns remain serious infections and psychiatric events (26). From a mechanistic perspective, belimumab does not inhibit the APRIL pathway, which may theoretically limit its impact on APRIL-dependent LLPCs. Telitacicept, by simultaneously targeting both BAFF and APRIL, could potentially modulate a wider range of pathogenic B-cell subsets, including those responsible for sustained aPL production. Although direct head-to-head comparisons regarding aPL reduction are currently lacking, this extended mechanistic profile offers a distinct and potentially complementary therapeutic approach. While this dual action may theoretically confer a broader immunosuppressive effect, the safety data from adult trials and our case experience have been reassuring, with infection rates appearing comparable to those of belimumab and without new or unexpected safety signals (27). The absence of adverse events, such as cytopenias or infusion reactions, in our patient during the follow-up period is encouraging. Nonetheless, the long-term safety of telitacicept in children, especially regarding its impact on humoral immunity and vaccination response, requires continued vigilance and larger prospective studies.

This case suggests that telitacicept may represent a promising therapeutic option for children with refractory SLE-APS, potentially enabling steroid reduction and sustained remission. However, these preliminary findings necessitate confirmation in larger, prospective studies. Future research should include multicenter randomized controlled trials to establish efficacy and safety, longer-term registries to monitor for rare adverse events, and biomarker studies to identify patients most likely to benefit from this targeted therapy.

## Conclusion

Enhanced clinical vigilance for thrombotic complications remains crucial in managing cSLE-APS. Currently, evidence regarding biologic therapies for cSLE-APS is limited, with targeted treatment strategies being particularly underexplored. To our knowledge, this represents the first reported case of successful cSLE-APS management using a telitacicept-based combination regimen. Our findings provide valuable clinical evidence to optimize biologic therapy approaches for this high-risk pediatric population, while addressing critical knowledge gaps in the treatment of autoimmune-mediated thrombotic disorders. This case advances the potential for personalized therapeutic strategies in cSLE-APS management. The primary value of this report is to describe a novel therapeutic approach, generate a hypothesis, and provide preliminary evidence to inform future larger-scale clinical studies. It is important to emphasize that this is a single-case report, and its findings cannot be generalized. The results and conclusions need validation in larger, prospective cohorts.

## Data Availability

The original contributions presented in the study are included in the article/supplementary material. Further inquiries can be directed to the corresponding author.
